# Are more diverse parts of the mammalian skull more labile?

**DOI:** 10.1002/ece3.2046

**Published:** 2016-03-04

**Authors:** Marta Linde‐Medina, Julia C. Boughner, Sharlene E. Santana, Rui Diogo

**Affiliations:** ^1^ Department of Orthopaedic SurgeryUniversity of CaliforniaSan FranciscoCalifornia; ^2^ Department of Anatomy & Cell BiologyUniversity of SaskatchewanSaskatoonSKCanada; ^3^ Department of BiologyBurke Museum of Natural History and CultureUniversity of WashingtonSeattleWashington; ^4^ Department of AnatomyHoward University College of MedicineWashingtonDistrict of Columbia

**Keywords:** Evolutionary rate, geometric morphometrics, Ornstein–Uhlenbeck model, Pagel's delta

## Abstract

Morphological variation is unevenly distributed within the mammalian skull; some of its parts have diversified more than others. It is commonly thought that this pattern of variation is mainly the result of the structural organization of the skull, as defined by the pattern and magnitude of trait covariation. Patterns of trait covariation can facilitate morphological diversification if they are aligned in the direction of selection, or these patterns can constrain diversification if oriented in a different direction. Within this theoretical framework, it is thought that more variable parts possess patterns of trait covariation that made them more capable of evolutionary change, that is, are more labile. However, differences in the degree of morphological variation among skull traits could arise despite variation in trait lability if, for example, some traits have evolved at a different rate and/or undergone stabilizing selection. Here, we test these hypotheses in the mammalian skull using 2D geometric morphometrics to quantify skull shape and estimating constraint, rates of evolution, and lability. Contrary to the expectations, more variable parts of the skull across mammalian species are less capable of evolutionary change than are less variable skull parts. Our results suggest that patterns of morphological variation in the skull could result from differences in rate of evolution and stabilizing selection.

## Introduction

Comparative studies have shown that different parts of the mammalian skull display different degrees of morphological variability, the face being generally more variable than the braincase, and the mandible more variable than the cranium (e.g., Marcus et al. 2000; Bennett and Goswami 2013; Figueirido et al. 2013). It is generally assumed that these differences in morphological variation largely reflect differences in lability, which can be widely defined as the propensity of a structure to evolve. It has been argued that this potential to evolve is influenced by the structural organization of the body part, commonly measured in terms of morphological integration and modularity (for a recent review, see Klingenberg 2013). Morphological integration and modularity refer to the capacity of different parts of a structure to covary – that is, to change in coordinated versus independent ways relative to one another, respectively. These concepts are not mutually exclusive; many structures may be integrated and still maintain a degree of independence (e.g., Porto et al. 2009; Klingenberg 2013). Modularity is generally interpreted as a mechanism by which the constraints associated with integration can be attenuated (e.g., Vermeij 1973; Liem 1980; Wagner and Altenberg 1996). However, under certain selective conditions (i.e., when the covariance pattern is oriented toward the direction of selection), integration could also act as a *line of least resistance* of evolutionary change, increasing morphological variation along certain directions (e.g., Schluter 1996; Marroig and Cheverud 2005; Goswami et al. 2014).

Differences in morphological variation among skull regions may result from differences in lability; however, there are at least two alternative explanations to this pattern: skull traits with the same capacity for evolutionary change can display different degrees of morphological variation if they evolved either *at different rates* (e.g., O'Meara et al. 2006; Goswami et al. 2014), or under *different selection regimes* (e.g., Butler and King 2004). The *different rates* scenario distinguishes between the potential of a structural organization to generate variation, which can define the morphospace potentially available to a trait, and the rate at which this variation is generated over time. That is, a trait that evolves at a high rate could display higher morphological variation than a trait that is more labile but evolves slowly, because the latter could have a larger, but unoccupied, potential area in morphospace (e.g., Hallgrímsson et al. 2009). Whereas structural organization and evolutionary rate could internally constrain trait evolution (i.e., via genetics/developmental processes) (e.g., Maynard Smith et al. 1985), the second alternative contemplates the possibility that a trait displays low variation not because of an internal constraint, but because it has been constrained externally (i.e., by stabilizing selection). These two alternative explanations are not mutually exclusive, and it is feasible for a trait to be simultaneously constrained both internally and externally, or a trait to have both low lability and low evolutionary rate, among other possibilities.

As a consequence of these varied factors, the breadth of scatter in morphospace could not be considered as an accurate estimate of trait lability. In other words, greater shape variation does not equate with greater capacity to evolve. Furthermore, “lability” is a species‐level term that, therefore, would not refer to the scatter in morphospace of a clade but rather to the evolutionary potential of a species. The evolutionary response of a species has been traditionally measured in quantitative genetics by Lande's equation (Lande 1979): *∆z = **G**β*, where ***G*** is an additive genetic variance/covariance matrix, *β* is a selection gradient representing those trait values with highest fitness, and *∆z* is the system's response to the selection gradient, that is, the resultant trait values after one generation. Hansen and Houle (2008) used Lande's equation to provide definitions of evolvability and other useful indexes that can be quantified and compared among species. These authors defined evolvability as the projection of the response vector on the selection vector. This definition explicitly captures the ability of a species to evolve in the direction of selection. The length of the response vector is called respondability and indicates how quickly the species responds to selective pressures (Hansen and Houle 2008).

Another index, flexibility, captures the ability of a species' response to align with the direction of selection, irrespectively of the magnitude of the response (Marroig et al. 2009). If a species' evolutionary response is closely aligned to the direction of selection, even if this species has low evolvability, it will have high flexibility (Marroig et al. 2009). Therefore, evolvability and flexibility capture discreet but relevant aspects of a species' response to selection. When the selection gradient (*β*) is unknown, these indexes can be estimated as their average under randomly generated selection vectors (Hansen and Houle 2008; Marroig et al. 2009).

Here, we aim to address the question, are more diverse parts of the mammalian skull more labile? We do so using a large sample, spanning all major extant mammalian clades, to quantify shape variation of different parts of the skull. We explore the existence of constraints and estimate rates of evolution of different parts of the skull by fitting our shape data to evolutionary models. We reconstruct the ancestral shapes of different skull parts at the root of the mammal phylogeny and apply the framework developed by Hansen and Houle (2008) and Marroig et al. (2009) to these reconstructed ancestral shapes to estimate their respondability, evolvability, and flexibility, which we use as proxies for lability. Combining estimations of constraint, rate of evolution, and lability, we provide an answer to this question.

## Material and Methods

### Data collection

**Figure 1:**
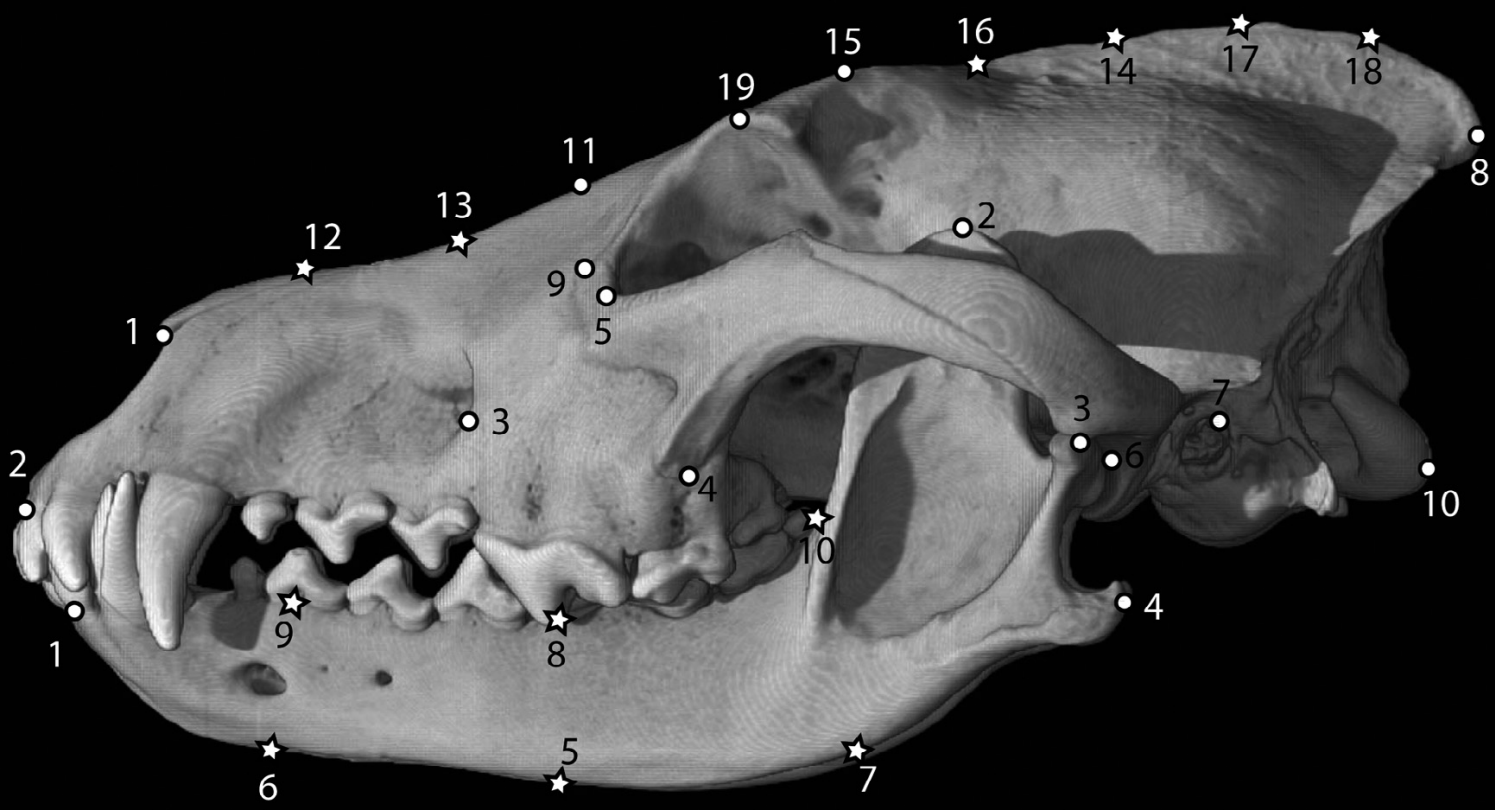
Shape data used in this study. Localization of the landmarks (circles) and semi‐landmarks (stars) used to define cranium and mandible shapes of mammals. Cranium landmarks 1, 2, 3, 4, 5, 6, 7, 8, and 9 corresponded, respectively, to landmarks 10, 11, 22, 23, 24, 26, 27, 7 and 33 previously defined by Marcus et al. (2000). Cranial landmarks 10, 11, 15, and 19 corresponded, respectively, to most posterior point of the occipital condyle, projection of landmark 9 on the facial contour, most anterior point of the contour of the cranial vault (behind the orbit) and point of maximum curvature of the orbit. In those cases where landmark 1 was behind landmark 9 (i.e., *Elephas maximus*,* Tapirus*,* Trichetus*,* Lagenorhynchus obliquus*,* Phocoena phocoena* and *Trusiops truncatus*), we recorded landmark 11 as the most posterior point of the facial contour, which is equivalent to the position of this landmark in other species. Mandible landmarks 1, 2, and 4 corresponded, respectively, to landmarks 1, 3, and 5 in Marcus et al. (2000). Mandible landmark 3 corresponded to tip of the condyloid process. The face and the braincase were defined by points [1–5, 11–13] and [6–10, 14–19], respectively (image downloaded from DigiMorph).

We compiled digital images (lateral view) of 467 crania and 207 mandibles representing all major mammalian lineages from open online sources: DigiMorph (University of Texas, USA), Mammalian Crania Photographic Archive (Dokkyo Medical University, Japan), Museum Victoria (Australia), Animal Diversity Web (University of Michigan, USA), Morphobank (S.U.N.Y., American Museum of Natural History, NY, USA), P.W. Lund's collection (Natural History Museum of Denmark), African Rodentia website (Royal Museum for Central Africa, Royal Belgian Institute of Natural Sciences and University of Antwerp), and Will's Skull Page (Table 1). Only photographs with a scale bar were included. For each species, we quantified skull shape recording the *xy* coordinates of a set of 13 landmarks and six semi‐landmarks (cranium), and four landmarks and six semi‐landmarks (mandible) using the tpsDig software v. 2.20 (Rohlf 2015) (Fig. 1). The number of homologous landmarks available to describe the shapes of these skull bones was dictated in large part by the use of lateral view images (the most available online resource) and the morphological diversity of the sample. We superimposed landmark configurations using Generalized Procrustes Analysis (GPA) to generate new sets of coordinates (i.e., Procrustes coordinates) that contain shape information. Semi‐landmarks were slid by minimizing bending energy (Gunz and Mitteroecker 2013). We computed Procrustes coordinates from separate Procrustes fits for the face and braincase. Comparisons between the cranium and mandible were carried out on a subset of 207 species.

**Table 1:** Number of species per taxon included in this study

Lineage	Order	Cranium	Mandible
Afrotheria	Afrosoricida	4	1
	Hyracoidea	1	1
	Macroscelidea	1	1
	Proboscidea	1	1
	Sirenia	2	2
	Tubulidentata	1	1
Euarchontoglires	Dermoptera	2	1
	Lagomorpha	6	1
	Primates	87	55
	Rodentia	120	20
	Scandentia	2	2
Laurasiatheria	Carnivora	92	43
	Cetartiodactyla	34	14
	Chiroptera	31	16
	Eulipotyphla	8	3
	Perissodactyla	8	3
	Pholidota	1	–
Marsupialia	Dasyuromorphia	10	6
	Didelphimorphia	8	4
	Diprotodontia	28	16
	Microbiotheria	1	1
	Notoryctemorphia	1	1
	Paucituberculata	2	2
	Peramelemorphia	5	5
Monotremata	Monotremata	2	2
Xenarthra	Xenarthra	9	5

### Data analysis

We distinguished between size‐dependent (e.g., allometry) and size‐independent shape variation by performing a multivariate regression of shape onto size and calculating the residuals from this regression. To account for the nonindependence of species (Felsenstein 1985), we included phylogenetic information onto morphospace using squared‐change parsimony (Klingenberg and Gidaszewski 2010). This mapping reconstructs the character states at the internal nodes by minimizing the total length of the tree. We then computed phylogenetic independent contrasts (PIC) (Felsenstein 1985; Klingenberg and Gidaszewski 2010). To test for allometry, we conducted a multivariate regression of PIC of Procrustes coordinates on PIC of *log* centroid size (Klingenberg and Marugán‐Lobón 2013). We determined the statistical significance of the allometric pattern by a permutation approach in which regression parameters were calculated after randomly reshuffling size and shape observations (10,000 iterations) (Klingenberg and Gidaszewski 2010). Here, we considered an allometric pattern as significant when the probability of randomly obtaining a regression vector that accounted for a higher percentage of shape variance was lower than 0.05. Where the regression was significant, we used the regression coefficients to calculate size‐corrected values of the original data (i.e., shape residuals) (Garland and Ives 2000; Klingenberg and Marugán‐Lobón 2013). We used these size‐corrected data to perform subsequent analyses.

We fitted the following evolutionary models to shape data: Brownian motion (BM), Ornstein–Uhlenbeck (OU), Early‐burst (EB), and delta (Felsenstein 1973; Pagel 1999; Butler and King 2004; Harmon et al. 2010). BM is an unconstrained, random model of evolution where species evolve along any direction of the morphospace at a constant rate (*σ^2^*) (Felsenstein 1973). In the OU model considered here, species evolve by BM but they are constrained toward a central point (*θ*); the parameter *α* measures the strength of this constraint (Butler and King 2004). Early‐burst is a time‐dependent model where the rate of evolution exponentially accelerates or decelerates through time. Low and high values of the rate change parameter, *ρ*, indicate early or late burst of morphological diversification, respectively (Harmon et al. 2010). In the delta model, evolutionary rate also changes through time. Similar to an EB model, low and high values of the delta parameter (*δ*) indicate that evolution has been concentrated closer to the root or the tips of the phylogenetic tree, respectively (Pagel 1999). We also fitted a combined evolutionary model based on the two models with the best‐fit values for each skull region (see below). The goodness‐of‐fit of each evolutionary model was assessed by the Akaike information criterion (AIC) (Akaike 1973). We selected the evolutionary model with the lower AIC score for each skull region. We estimated evolutionary rates of different parts of the skull through $\sigma_{\mathrm{mult}}^{2}$, the average evolutionary rate of a trait along each dimension of morphospace (Adams 2014; Goolsby 2015).

Estimations of respondability, evolvability, and flexibility (Hansen and Houle 2008; Marroig et al. 2009) were computed on mean standardized *P*‐matrices of the ancestral reconstruction of different parts of the skull shape at the root of the mammal phylogeny (Revell 2012). The framework developed by Hansen and Houle (2008) is based on *G*‐matrices; however, *P*‐matrices could also be used if they show strong similarity to *G*‐matrices, as it has been demonstrated among mammalian groups (e.g., Cheverud 1988, 1996). The indexes of the ancestral shape for each skull part were estimated using the random skewers method (Cheverud and Marroig 2007): A set of 1000 randomly generated selection vectors were applied to a specific P‐matrix to obtain 1000 response vectors; respondability was calculated as the average length of the simulated response vectors, evolvability, as the average of the projection of the simulated response vectors to the corresponding selection vectors, and flexibility, as the average correlation between simulated responses and their corresponding selection gradients (Hansen and Houle 2008; Marroig et al. 2009).

We computed GPA in R (R Core Team 2015) using *gpagen* in GEOMORPH v. 2.1.6 (Adams and Otárola‐Castillo 2013). We used MorphoJ v. 1.06d (Klingenberg 2011) to compute multivariate regressions and PICs. Ancestral reconstructions of skull shape were performed in R using *fastAnc* in PHYTOOLS v. 0.4.60 (Revell 2012). Mean standardized *P*‐matrices and estimations of respondability, evolvability, and flexibility were computed in R using *meanStdG* in EVOLVABILITY v. 1.1.0 and *MeanMatrixStatstics* in EVOLQG v. 0.2.1, respectively (Melo et al. 2015). Fitting of evolutionary models and tree transformation were carried out in R using *rate.mult* in PHYLOCURVE v. 1.3.0 and *rescale* in GEIGER v. 2.0.6 (Harmon et al. 2008), respectively. Ancestral reconstructions were computed using the three phylogenetic trees provided by Bininda‐Emonds et al. (2007). Other analyses were based on the best‐dates phylogenetic tree (Bininda‐Emonds et al. 2007). Polytomies were resolved using the *multi2di* function in APE v. 3.3 (Paradis et al. 2004).

## Results

### Face and braincase

Size explained 2.3% (*P* < 0.0001) and 0.6% (*P* < 0.05) of shape variation in the face and braincase, respectively. In terms of average squared Procrustes distance (in tangent space), face shape was two times more diverse than braincase shape (Table 2). For these skull regions, a combined model showed the lowest AIC score, in which evolutionary change was concentrated toward the tips of the phylogenetic tree (delta model), and there was a central tendency constraining shape evolution (OU model). Delta parameter estimates differed between the face and the braincase, indicating that their relatively recent acceleration of morphological change occurred at different time points (i.e., the braincase appears to have undergone accelerated morphological change more recently than the face) (Table 2). The *α* parameter was the same for both face and braincase shapes, indicating that both regions have experienced a similar magnitude of constraint.

**Table 2:** Values of evolutionary parameters (*δ* and *α*), average squared Procrustes distance (*P^2^*), rate of evolution ($\sigma_{\mathrm{mult}}^{2}$), respondability ($\overset{¯}{r}$), evolvability ($\overset{¯}{e}$), and flexibility ($\overset{¯}{f}$) for different parts of the mammalian skull

	*δ*	*α*	*P^2^*	$\sigma_{\mathrm{mult}}^{2}$	$\overset{¯}{r}$	$\overset{¯}{e}$	$\overset{¯}{f}$
Face	1.99	0.0052	0.080	5.61E^−05^	2.17E^−04^	7.18E^−05^	0.24
Braincase	2.43	0.0053	0.033	1.95E^−05^	3.83E^−03^	1.01E^−03^	0.17
Cranium	1.37	0.0046	0.042	1.45E^−05^	3.44E^−04^	6.95E^−05^	0.14
Mandible	–	0.0073	0.031	2.87E^−05^	9.81E^−04^	2.71E^−04^	0.19

According to $\sigma_{\mathrm{mult}}^{2}$ value, face shape evolved twofold faster than braincase shape. In contrast to face shape, braincase shape showed markedly higher respondability and evolvability (Table 2). Although facial shape was less evolvable in terms of magnitude, it displayed a higher flexibility than braincase shape, that is, a higher capacity to orient in the direction of selection (Table 2). These results suggest that the face is not more variable because it has been more labile, but because it has evolved at a faster rate than the braincase. The low variation in braincase shape despite its high lability cannot be attributed to stronger stabilizing selection (i.e., external constraint) because both the braincase and facial skeleton appear to have undergone a similar magnitude of constraint according to the results found here.

### Cranium and mandible

Size explained 2.6% (*P* < 0.0001) and 2.5% (*P* < 0.001) of shape variation in the cranium and mandible, respectively. Cranium shape showed higher variation than mandible shape (Table 2). A combined model of cranium shape evolution had a best‐fit value and described a recent increase in evolutionary rate (delta model) and presence of constraint (OU model). Mandible shape evolution was best described by an OU model (Table 2). The *α* parameters indicate that the strength of constraint has been stronger on the shapes of the mandible than of the cranium (Table 2).

Mandible shape has evolved at a faster rate than cranium shape. Mandible shape also showed a higher degree of both respondability and evolvability than braincase shape. However, both mandible and cranium presented similar flexibility indexes (Table 2). Although mandible shape has been more labile and has evolved at a faster rate, it is less variable than cranium shape. Our results suggest that this pattern of morphological diversity could be due to the existence of a stronger external constraint on mandible shape evolution (as measured by *α* parameter) (Table 2).

## Discussion

It is commonly thought that differences in morphological diversity among regions of the mammalian skull mainly reflect differences in lability (i.e., their propensity to evolve). For example, the lower scatter in morphospace of cranium shape relative to mandible shape in durophagous carnivorans has been interpreted as a limited capacity of the cranium to evolve toward particular feeding adaptations due to the higher structural complexity and multi‐functionality of this skull part (Figueirido et al. 2013). In another comparative study of the cranium (Bennett and Goswami 2013), the reduced area of morphospace occupied by marsupials in comparison with placentals has been interpreted as the consequence of the early ossification of the oral region, which could act as a developmental constraint on subsequent Marsupial cranial evolution. Contrary to these expectations, here we have shown that less variable skull regions could be more labile than highly variable regions. Our results suggest that observed patterns of morphological variation in the mammalian skull could be the result of differences in rates of evolution in the face versus the braincase, and of external constraints on the cranium versus the mandible.

Marroig and Cheverud (2005) reported that a high portion of cranium shape variation across New World monkeys was size‐related and proposed that changes in cranial morphology originated as by‐products of selection for body size. According to this hypothesis, cranial allometry could act as a *line of least resistance* that facilitates evolutionary changes in the cranial region (Marroig and Cheverud 2005). The ubiquity of cranial allometry in other eutherians and metatherian groups, in combination with the wide range of body sizes evolved by mammals, have led other authors to suggest that body size could influence the evolution of cranial diversity across Mammalia (Cardini and Polly 2013; Cardini et al. 2015). Our broad, phylogenetic comparative analysis has shown, however, that size explains 2.6% of cranium shape variation across all mammals, and therefore, its relevance in cranial diversification might not be generalizable for the entire class.

In a past test of the relevance of strength of modularity to cranial evolution, Goswami and Polly (2010) compared morphological disparity between weak and strongly integrated cranial modules in carnivorans and primates. The authors hypothesized that if strength of modularity has favored cranial evolution, strong modules (i.e., those with high within‐module covariation) would display higher levels of morphological disparity and vice versa. However, for the most part, the authors found no significant difference in morphological disparity between cranial modules defined a priori (Goswami 2006) and randomly selected modules. Contrary to expectations, in the few cases where morphological disparity did significantly differ, the results supported the constraint model, that is, strong modules had lower morphological disparity than expected. The authors concluded that there is not a single rule for the role of modularity on macroevolution (Goswami and Polly 2010).

Here, we would like to stress that the role of structural organization on morphological diversification does not necessarily depend on the *strength* of trait covariation, but on the *alignment* of trait covariation with the direction of selection. That is, both weak and strong modules could facilitate morphological evolution if their patterns of trait covariation align to the direction of selection, and both strengths of modules could constrain evolution if they are misaligned to this direction. Furthermore, as our analysis shows, the potential of a given structural organization to favor evolutionary change is not accurately and comprehensively assessed only by measures of morphological variation because other factors (i.e., rate of evolution, external selective demands) can affect trait variation. Instead, assessment of lability is strongest when based on estimates of respondability, evolvability, and/or flexibility (Hansen and Houle 2008; Marroig et al. 2009). For example, Marroig et al. (2009) explored the role of cranial organization in mammals by correlating indexes of morphological integration and modularity with indexes of respondability, evolvability, and flexibility. The authors found a significant relationship between cranial organization and flexibility that would support the existence of a general rule for the role of integration and modularity on the evolution of the mammalian skull (Marroig et al. 2009).

As previously stressed by Porto et al. (2009), although the relevance of broad comparative analyses of morphological variation is widely accepted, these studies are scarce in mammals. We hope that the present study helps to fill this gap of information and further improve our understanding of skull evolution.

## Conflict of Interest

None declared.
